# Fabrication of Bentonite–Silica Sand/Suspended Waste Palm Leaf Composite Membrane for Water Purification

**DOI:** 10.3390/membranes10100290

**Published:** 2020-10-16

**Authors:** Saad A. Aljlil

**Affiliations:** National Center for Water Treatment and Desalination Technology, King Abdulaziz City for Science and Technology, P.O. Box 6086, Riyadh 11442, Saudi Arabia; saljlil@kacst.edu.sa

**Keywords:** extrusion, ultrafiltration membrane, bentonite clay, silica sand, cetyl trimethyl ammonium bromide, water purification

## Abstract

In this study, a method for fabricating tubular ceramic membranes via extrusion using economical and locally available bentonite–silica sand and waste palm leaves was developed as a tool for conducting the necessary task of purifying water polluted with oil and suspended solid materials produced via various industrial processes. The developed tubular ceramic membranes were found to be highly efficient at separating the pollutants from water. The properties of the fabricated membrane were evaluated via mechanical testing, pore size distribution analysis, and contact angle measurements. The water contact angle of the fabricated membrane was determined to be 55.5°, which indicates that the membrane surface is hydrophilic, and the average pore size was found to be 66 nm. The membrane was found to demonstrate excellent corrosion resistance under acidic as well as basic conditions, with weight losses of less than 1% in each case. The membrane surface was found to be negatively charged and it could strongly repulse the negatively charged fine bentonite particles and oil droplets suspended in the water, thereby enabling facile purification through backwashing. The obtained ceramic membranes with desirable hydrophilic properties can thus serve as good candidates for use in ultrafiltration processes.

## 1. Introduction

Globally, the oil production industry is responsible for the production of approximately 210 million barrels of oil-contaminated water per day, which costs approximately USD 45 billion to eliminate from the environment [1]. Saudi Arabia is currently facing the challenge of recycling large amounts of this polluted water to reduce its negative environmental effects—particularly on groundwater resources—which threaten both the country’s aquatic wealth and human health [2]. Concurrently, the necessity of obtaining unpolluted water has increased in recent years as water resources dwindle and costs rise [3]. Finally, the ability to eliminate suspended solids from industrial wastewater serves as a significant benchmark of overall water treatment quality [4]. One efficient method for the removal of suspended materials is sedimentation [4]. Another method relies on the use of ceramic membranes, which are usually fabricated from expensive oxides such as alumina, zirconia, and titania [5,6]. Scientists continue to search for new, inexpensive, and safe materials for the fabrication of such ceramic membranes. These membranes possess properties amenable to the purification of polluted water, including high thermal stability and mechanical strength, and they can be cleaned easily to prevent fouling [7]. Microfiltration, ultrafiltration, and nanofiltration membranes are widely recognized for their ability to yield clean water through the rejection of suspended as well as dissolved materials. Zaidi et al. [8] investigated microfiltration and ultrafiltration membranes for the elimination of oil and solid particles from wastewater to obtain reusable water. Bilstad and Espedal [9] studied the efficiency of microfiltration and ultrafiltration membranes in a pilot oilfield plant to remedy wastewater through the removal of oil. They determined that ultrafiltration membranes performed well in terms of reducing the total hydrocarbon content and dissolved material load. Ceramic membranes that can effectively purify water of oil and suspended materials via a tubular ceramic microfiltration process [10] have been developed on the basis of the use of natural raw materials, primarily inorganic nonmetallic crystalline materials such as clay [11], magnesite [12], and dolomite [13]. Majouli et al. [14] fabricated tubular microfiltration membranes from Moroccan perlite for water purification. Other researchers have employed low-cost materials such as fly ash [15] and industrial waste [16] as fillers for the fabrication of ceramic membranes. Ben Amar and Oun [17] used Tunisian mud as a low-cost material for the fabrication of tubular ceramic ultrafiltration membranes for removing pollutants from wastewater. These membranes were obtained via the slip-casting technique followed by sintering at 650 °C. With a pore size of 11 nm, the membranes demonstrated a permeability of 90 L/h·m^2^·bar and a chemical oxygen demand (COD) pollutant removal of 90%. Hubadillah et al. [18] used kaolin as a low-cost ceramic raw material for the fabrication of a ceramic hollow fiber membrane and achieved improved mechanical strength and excellent performance in terms of pollutant removal from wastewater. Issaoui and Lionel [19] have reported the fabrication of commercialized ceramic membranes based on expensive materials such as cordierite, titania, zirconia, and silicon carbide for various industrial applications. Therefore, to decrease the cost, efforts have been dedicated toward utilizing low-cost materials such as natural clay, apatite powder, dolomite, kaolin, bauxite, and mineral coal fly ash for the fabrication of ceramic membranes exhibiting good performance for waste water and pollutant treatment. Kakali and Pugazhenthi [20] focused on using lithium aluminosilicate for the fabrication of a ceramic membrane via the slip-casting method, using starch as the pore-forming material for removing bacteria such as Escherichia coli, Bacillus cerues, and Enterococcu faecalis from wastewater; they achieved good performance in terms of removal of bacteria from wastewater. In this study, we aimed to use widely available, economical, and natural raw materials such as bentonite, silica sand, and suspended waste palm leaves, and cetyl trimethyl ammonium bromide (CTAB) to fabricate a tubular ultrafiltration ceramic membrane for water purification applications.

### Significance and Novelty of This Work

This work is significant in that it presents the successful fabrication of a new tubular ceramic membrane for water purification using economical, locally available materials such as bentonite clay, silica sand, and suspended waste palm leaves. By means of using economical and ecologically friendly bentonite as a plasticizer and silica sand powder from Saudi Arabia, cetyl trimethyl ammonium bromide (CTAB) as a binder, and waste palm leaves as the pore-forming material, a ceramic membrane filtration system for water purification and wastewater treatment was fabricated via an extrusion technique. Our results suggest that the use of waste palm leaves as a pore-forming material decreases the cost of the membrane fabrication process. The synthesized composite membrane represents a cost-efficient solution for conducting filtration processes for treating oily wastewater, removing suspended materials from wastewater, and for water pretreatment in seawater desalination plants.

The novelty of this work lies in the fabrication of a highly effective composite membrane through the combination of inorganic materials (bentonite clay with silica sand) and waste palm leaves to produce a porous membrane. By means of using a constant-pressure fluid–fluid porometer, the average pore size of the membrane was determined to be 66 nm, which indicated that ultrafiltration could be achieved without the use of a coating layer. The nanometer-scale pore size was achieved using waste palm leaf powder as a pore former via a process wherein palm leaf paste was first sintered and then burned. As the average pore size of the membrane of 66 nm is within the pore size range known to be suitable for ultrafiltration membranes, which ranges up to 100 nm, ultrafiltration can be directly achieved without the use of a coating layer.

To the best of our knowledge, all previous approaches on the development of ultrafiltration membranes have involved the fabrication of membranes as microfiltration substrates followed by the application of an interlayer and filtration coating layers. The new membrane fabricated in this study is also novel in terms of the waste palm leaves used as a pore-forming material. To the best of our knowledge, no studies have employed CTAB as a binder; most used carboxymethyl cellulose (CMC), a conventional binder. CTAB is a positively charged surfactant that is attracted to negatively charged membranes; herein, the fabricated membrane comprises mainly of silica, which has an inherent negative charge.

## 2. Materials and Methods

### 2.1. Raw Materials

**Figure 1:**
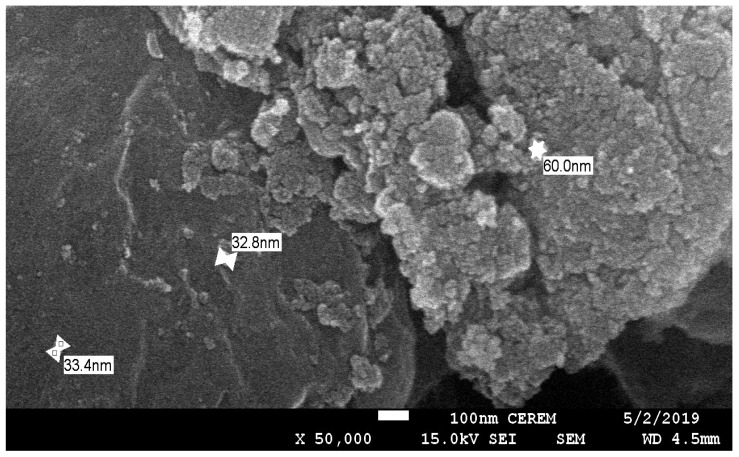
Scanning electron micrograph of treated palm leaves (Magnification = 50,000×).

Silica sand powder possesses beneficial properties, including a high degree of hardness and stability when it is exposed to chemicals. SiO_2_ is the main constituent of silica sand powder. The powder, with particle sizes below 100 µm, used in this study was obtained from the Biyadh Factory located close to the city of Riyadh, Saudi Arabia. The palm leaf waste material used for forming pores in the membrane was obtained from the Alhassa region of Saudi Arabia. The palm leaves were ground using a ball mill to a powder with a particle size of less than 65 μm, following which the powder was mixed with distilled water and shaken for 48 h. The obtained mixture was then subjected to ultrasonication (SFX550 Model, Sonifier, Mexico) for 40 min at an output power of 550 W. After ultrasonication, the formed colloidal suspension was centrifuged at 3500 rpm for 15 min. The resulting supernatant was dried to obtain a powder with a particle size of less than 43 nm, which was used to prepare a pore-forming paste. A scanning electron micrograph of the sample is shown in Figure 1. Further, CTAB, used herein as a binder, was obtained from Aldrich, USA. Saudi bentonite clay obtained from the Khulays Mine was crushed and milled to a mean particle size of less than 100 µm, cleaned repeatedly with distilled water, and then, dried in a vacuum oven for 24 h. The chemical compositions of the Saudi silica sand powder and bentonite clay were analyzed via X-ray fluorescence (Element Analyzer model JSX-3201, JEOL, Germany), and the results are listed in Table 1 and Table 2, respectively.

### 2.2. Membrane Fabrication

**Figure 2:**
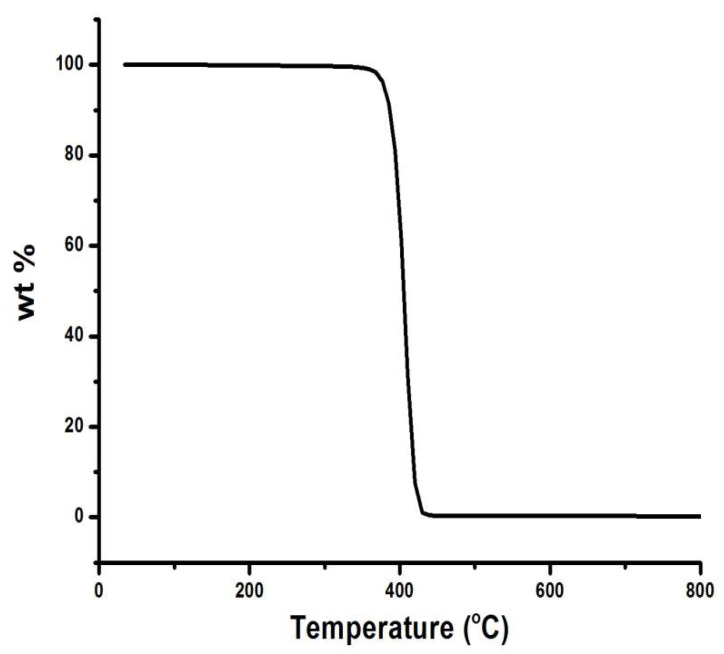
Thermogravimetric of waste leaves.

**Figure 3:**
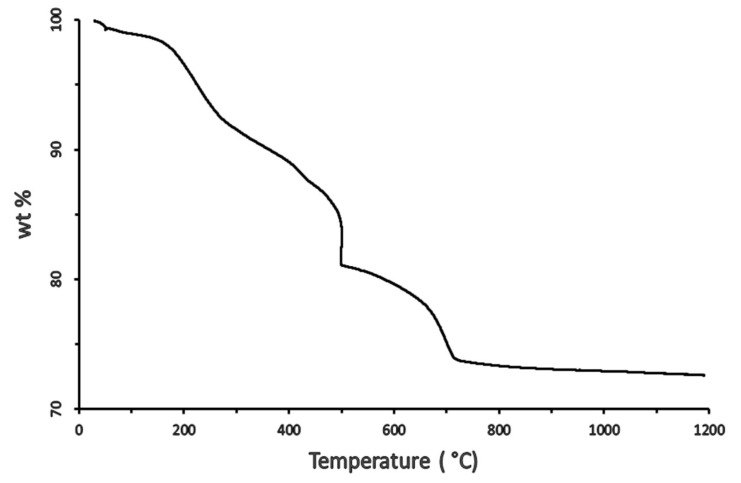
Thermogravimetric of the paste of the ceramic membrane.

**Figure 4:**
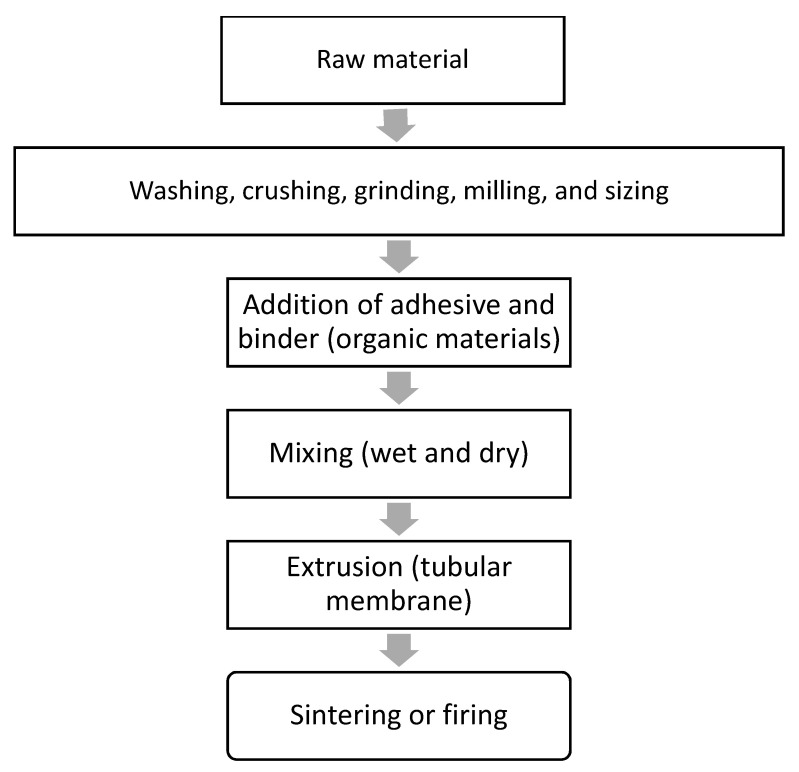
Flow diagram for fabrication of ceramic membrane.

The raw materials used to fabricate the membrane are as follows: 63.29 wt.% Saudi bentonite clay as a plasticizer, 31.65 wt.% Saudi silica sand, 3.17 wt.% CTAB (Aldrich, USA) as a binder, and 1.89 wt.% suspended waste palm leaf powder as a pore former. The powdered mixture of these materials was blended with 300 mL of water to create a wet paste for extrusion. Saudi bentonite clay was included as a plasticizer to promote plasticity and decrease the brittleness of the membrane during its extrusion. The bentonite clay was crushed using a planetary ball mill (Changsha Deco Equipment Co, Changsha, China) for 3 h at a speed of 250 rpm to produce a fine powder (particle size < 100 μm). The raw materials were dry-mixed using a z-mixer (Z-blade mixer, Winkworth, UK) for 2 h, following which a specific amount of water was gradually added during wet mixing until the plasticity of the paste was satisfactory, with negligible or minimal sticking of the paste to the inner wall of the mixing chamber. For production of the ceramic membrane, the paste was placed in an extruder cylinder and forced through a die using a piston. The dimensions of the extruded tubes were controlled using a die constructed to produce elongated individual tubes of the membrane with a constant cross-section. A drying process at 25 °C (room temperature) was then applied to the produced membrane using wooden grooves and a plastic film covering, and this process lasted 3 d. To prevent the cracking of the membrane during the drying process, the ceramic materials were consolidated by heating at a temperature below the melting point (1350 °C), at which individual particles would diffuse toward neighboring particles. The sintering process was performed in two stages: first, all the organic materials in the membrane were gradually burned out by increasing the temperature from 25 °C up to 500 °C at a rate of 2 °C/min to create pores in the fabricated membrane. Thermogravimetric analyzer (TGA 801, LECO, USA) of the waste leaves is critical for elucidating the sintering process (Figure 2). It is evident from Figure 2 that the waste leaves are burned completely at 450 °C. Subsequently, the membrane was subjected to densification by sintering, for which the temperature was increased from 500 °C to 950 °C at a rate of 3 °C/min for 3 h. The TGA graph of the paste is shown in Figure 3. From Figure 3, it is evident that the mixed material paste of the ceramic membrane is burned completely at 850 °C. The membrane sintering process was conducted by placing the membrane horizontally in an electric furnace (Nabertherm) and firing under normal atmospheric conditions. A flow diagram for the ceramic membrane fabrication process is shown in Figure 4.

### 2.3. Wastewater Preparation and Characterization

The effectiveness of the fabricated membrane was assessed via its application separately in the treatment of two wastewater samples: an artificial emulsion of oil (ultrapure paraffin oil) in water and a bentonite clay suspension of water with high solid content. The artificial emulsion was prepared by mixing paraffin oil and distilled water for 30 min using an agitator at a speed of 1300 rpm to produce a stable white emulsion that could remain so for several days under gravitationally induced separation forces. The emulsion was then transferred to a feed tank, in which an agitator was run at 1300 rpm to keep the emulsion stable. The droplet size distribution was measured using a laser analyzer (SALD-30 IV, Longcai Advanced Materials, China). The charge of the oil droplets in the emulsion was also characterized via measurement of the zeta potential using the Zetasizer (Malvern Nano ZS, Malvern Panalytical Ltd., UK). To determine the effectiveness of the fabricated membrane in terms of removing pollutants from the wastewater, the turbidity (NTU) was measured. To further evaluate the filtering capability of the membrane, the process described above was applied in the treatment of a suspension of very fine bentonite clay (particle size < 65 µm) in distilled water prepared using a magnetic stirrer, and the zeta potential of the fine bentonite clay particles was obtained using the Zetasizer (Malvern Nano ZS). The pore size distribution of the membrane was determined using a constant-pressure fluid–fluid porometer (IFTS advanced fluid-fluid porometer, Institut de la Filtration et des Techniques Séparatives, France).

### 2.4. Ultrafiltration Test with the Membrane

**Figure 5:**
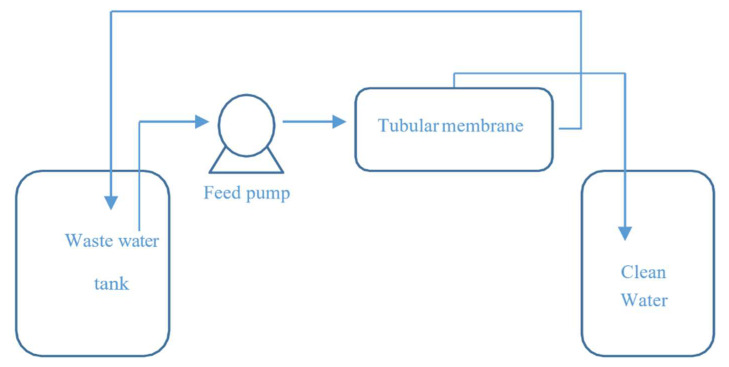
Schematic of experimental filtration.

The experimental setup shown in Figure 5 was then used to assess the water purification performance of the membrane for two wastewater mixtures—an oil-in-water emulsion and a suspension of fine bentonite powder in water. In each case, the feed was circulated through the fabricated tubular membrane (internal diameter of 0.46 cm, length of 19 cm, and surface area of 0.002744 m^2^) at an input flow rate of 55 L/h, input temperature of 25 °C, and a specified cross-flow pressure. The feed tank included an agitator to maintain the suspension and emulsion in stable states. The water permeates were collected in a clean water tank and weighed using a balance to calculate the water permeate flux using the following relation:

*J* = *V*/*A* × *t*, (1)

where *J* is the flux rate in L/m^2^·h, *V* is the collected water permeate volume in m^3^, *A* is the surface area of the fabricated membrane in m^2^, and *t* is time in h.

The turbidities of the water permeate and water in the feed tank were measured using a turbidimeter (Hach Company, USA), and these values were used to calculate the oil rejection (*R*%):

*R*% = [1 − (*C*_p_/*C*_f_)] × 100, (2)

where *C*_f_ (NTU) is the turbidity of the feed, and *C*_p_ (NTU) is turbidity of the water permeate.

The water permeability of the fabricated membrane was then calculated using the formula developed by Kumar et al. [21]:

J = *K*_p_ × Δ*P*, (3)

where *K*_p_ is the water permeability of the fabricated membrane (L/m^2^·h·bar), *J* is the water flux rate (L/m^2^·h), and Δ*P* is the difference in pressure between the two sides of the membrane (bar).

## 3. Results and Discussion

### 3.1. Characterization of the Wastewater and Fabricated Membrane

**Figure 6:**
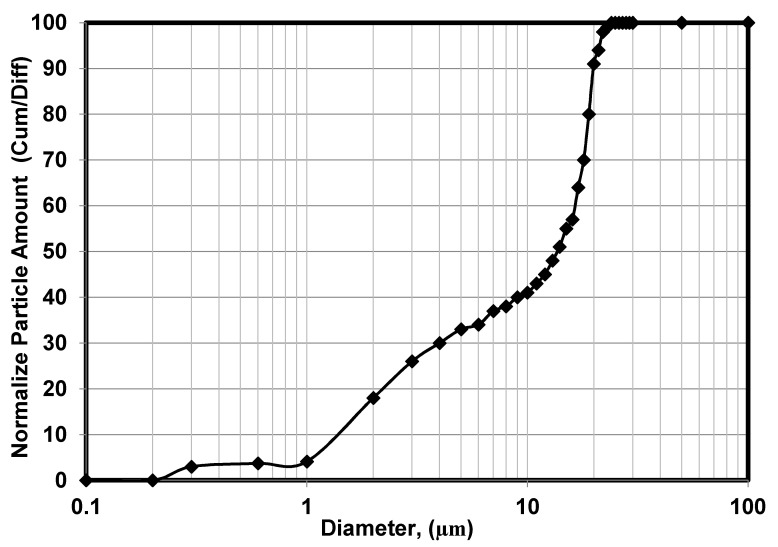
Oil droplet size distribution of artificially produced oil-in-water emulsion.

**Figure 7:**
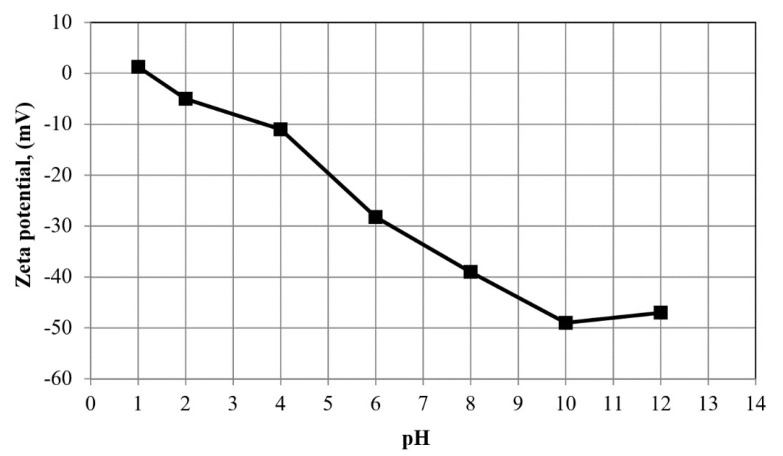
Zeta potential of oil droplets as a function of pH.

**Figure 8:**
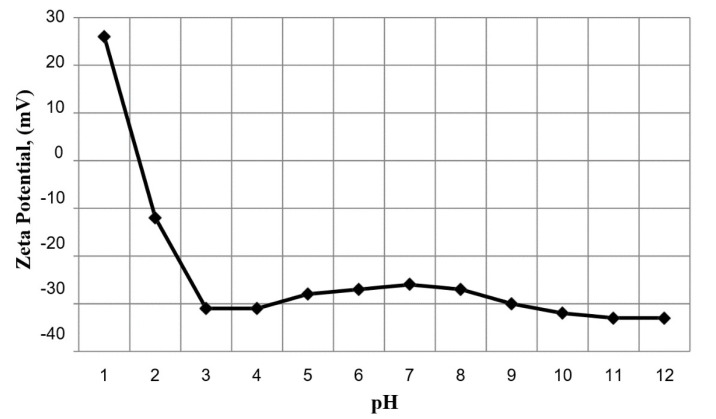
Zeta potential of fine bentonite clay as a function of pH.

**Figure 9:**
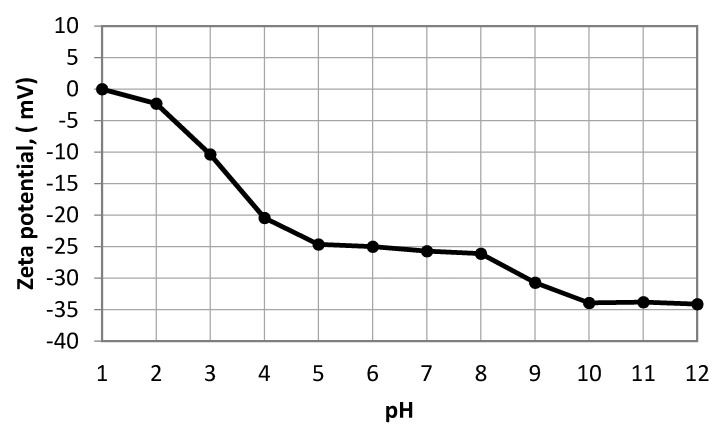
Zeta potential of fabricated membrane as a function of pH.

**Figure 10:**
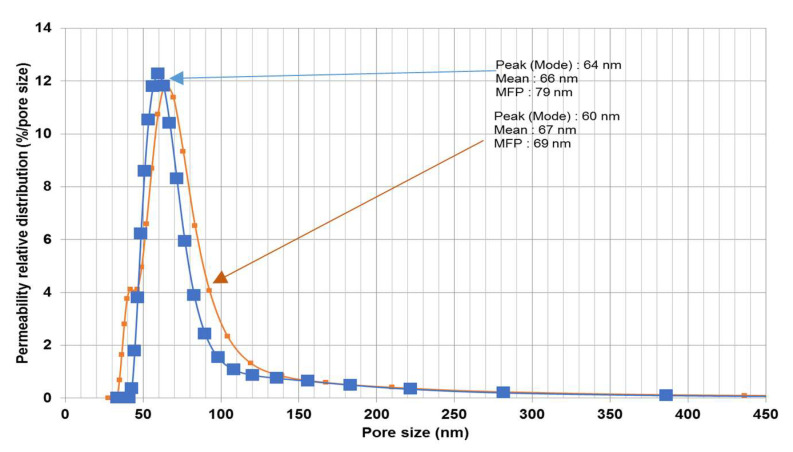
Pore size distribution of fabricated membrane.

The oil droplet size distribution determined using the laser analyzer (SALD-30 IV) is shown in Figure 6. The wastewater oil droplets had an average size of 5.87 μm. The zeta potential of the oil droplets in the emulsion was also measured as a function of pH (see Figure 7). The isoelectric point (PI) of the oil droplets corresponded to a pH of approximately 1.57, above which the zeta potential became negative. The measured zeta potential and droplet size distribution were consistent with results reported previously [22]. The zeta potential of a water suspension of the fine bentonite clay powder as a function of pH is shown in Figure 8. It is seen that the PI point corresponded to pH = 1.5, above which the surface charge became negative. The performance of a ceramic membrane is determined by its surface characteristics, pore size distribution, and mechanical properties. As discussed in Section 3.3, the effectiveness of our fabricated membrane in terms of eliminating fouling materials during backwash can be understood in terms of the well-known mutual electrostatic repulsive property of opposite charges with identical magnitudes. Further characterization of the membrane via the three-point flexural strength technique (crosshead speed of 0.5 mm/min, Shimadzu) to determine its mechanical properties revealed a sufficiently high bending strength (51.53 MPa; the stress–strain relationship of the ceramic membrane is linear). Further, a contact angle of 55.5° was determined via the thin layer wicking method, involving measurement of the contact angle of porous membranes [23], which indicates that the membrane is hydrophilic, and the zeta potential of the fabricated membrane as a function of pH is shown in Figure 9. It is seen that the PI point corresponded to pH = 1.5, above which it became negative. Figure 10 shows the pore size distribution obtained using a constant-pressure fluid–fluid porometer. The average membrane pore size of 66 nm indicates that the membrane can be used for ultrafiltration without the use of a coating layer.

### 3.2. Evaluation of the Membrane Performance

#### 3.2.1. Resistance to Chemical Corrosion

The stability of the membrane was evaluated in corrosive media with extremely high acidity and basicity to determine the membrane’s resistance to chemical corrosion. Mass losses after immersion at 25 °C for 7 d in HCl and NaOH solutions at pH = 1.5 and 13, respectively, were determined to be 0.76 and 0.12 wt.%, respectively, indicating a mass loss of less than 1% in each case. These results indicate near-perfect membrane resistance to the alkaline solution relative to the results obtained by Dong et al. [15,24] for alumina and cordierite membranes. A similar behavior was observed in the acidic solution. In highly concentrated alkaline solutions, alumina and cordierite membranes exhibit relatively poor corrosion resistances because more hydroxide ions react with these membranes in such cases, causing a significant increase in mass loss relative to that in the case involving our membrane. This is because the membranes have a very high content of alumina and cordierite in comparison with those in our membrane. The reaction between Al_2_O_3_ and the hydroxide ion is shown below:

Al_2_O_3_ + OH^−^ → AlO_2_^−^ + H_2_O

#### 3.2.2. Evaluation of the Membrane Performance Using Artificially Produced Wastewater

**Figure 11:**
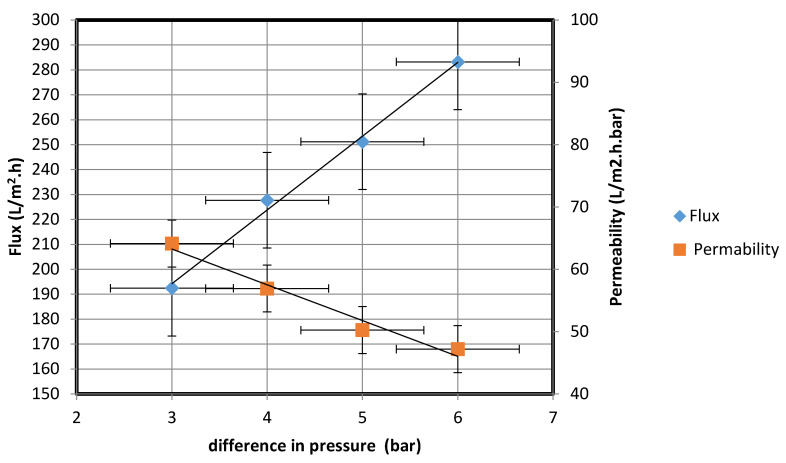
Water flux rate and permeability of fabricated membrane at different pressures.

By means of the experimental setup in Figure 5, the water permeate flux rate of a mixture of ultra-pure paraffin oil and distilled water (62.8 NTU) was calculated at pressures of 3, 4, 5, and 6 bar. In each case, the water permeate was collected after 4 h, and the water flux rate and water permeability of the fabricated membrane were calculated using Equations (1) and (3), respectively. The results are shown in Figure 11.

Figure 11 shows that the flux rate increased with the difference in the pressure between the two sides of the membrane; the flux rate increased from 192.41 to 283.20 L/m^2^·h upon the pressure difference being increased from 3 to 6 bar. The results could be fitted well with Darcy’s law, according to which water flow through a purification system is proportional to the hydraulic gradient [25]. The water permeability, plotted as a function of pressure difference shown in Figure 11, reveals that a high degree of water permeability was achieved at a low operating pressure; these results are similar to those obtained using a commercial seawater reverse osmosis system [26]. From the data in Figure 11, the standard deviation is 58.12, while the mean is 190.89; the low standard deviation implies the reliability of the data. Moreover, the 90% confidence level is in the range (238.7, 143.1). Therefore, we are 90% confident that the true mean (190.89) lies between 238.7 and 143.1. The margin of error is small.

The water permeate flux rate of a mixture of ultra-pure paraffin oil and distilled water (62.8 NTU) was then measured at a pressure of 5 bar. The operating pressure of the ultrafiltration membrane is in the range of 4–7 bar, according to industrial standards [27]. Therefore, 5 bar as the operating pressure was used to obtain a good flux rate.

**Figure 12:**
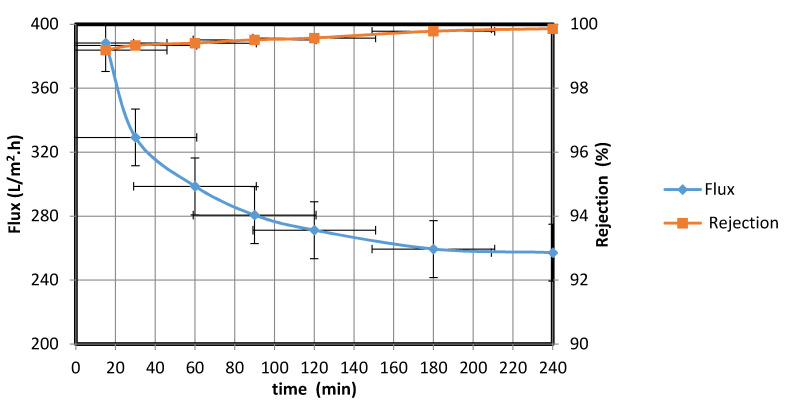
Water/synthetic oil flux rate through membrane and percentage rejection by fabricated membrane at different times.

The water permeate was collected at different intervals, and the water flux rates and percentage rejections were calculated using Equations (1) and (2), respectively (Figure 12).

Figure 12 shows that the water permeate flux rate decreased over the course of 4 h from 388.24 to 257.19 L/m^2^·h. This behavior possibly resulted from the increase in the amount of oil trapped in the membrane as the filtration progressed; the trapped oil decreased the membrane area available for water transfer. When the oil droplet size is larger than the membrane pore size, membrane fouling could occur, leading to a decline in the flux rate. Kumar et al. [21] observed a similar behavior when the droplet size exceeded the pore size; accumulation of a deposited layer on the membrane resulted in pore blockage and a decrease in the water permeate flux rate. To overcome this blockage, an effective method for cleaning the fouled membrane surface is required.

From the data in Figure 12, the standard deviation is 38.30, while the mean is 310.79. The low standard deviation proves the reliability of the data. Moreover, the 90 % confidence level is 334.60, 286.97. We are 90% confident that the true mean lies between 334.60 and 286.97 and the margin of error is small.

#### 3.2.3. Real Case Study Using Water Obtained from the Aramco Company

**Figure 13:**
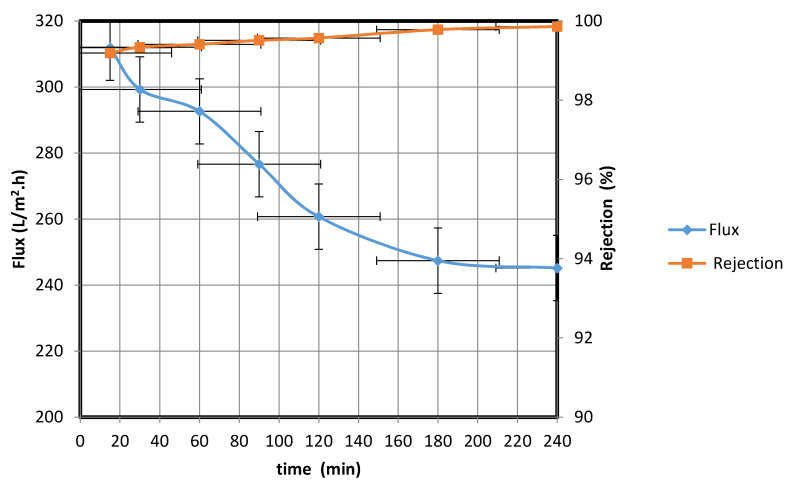
Flux rate of a real oil-contaminated water sample through fabricated membrane and percentage rejection at different times.

The water permeate flux rate was measured at a pressure of 5 bar for oil-contaminated wastewater samples (77.4 NTU), obtained from Aramco’s SFNY and ZULF wells, used as the feed solutions. The water permeate was collected after different time intervals, and the water flux rate and percentage rejection were calculated using Equations (1) and (2), respectively (Figure 13).

Over the course of the experiment, the oil rejection rate increased from 99.19 to 99.86%, while the water flux rate declined as a result of the deposition of oil droplets on the membrane surface and the increase in cake thickness on the surface. From the data in Figure 13, the standard deviation is 19.42, while the mean is 285.12. The low standard deviation proves the reliability of the data. Moreover, the 90 % confidence level is 297.19, 273.05. Therefore, we are 90% confident that the true mean lies between 297.19 and 273.05 and the margin of error is small.

#### 3.2.4. Removal of Suspended Materials from Wastewater

**Figure 14:**
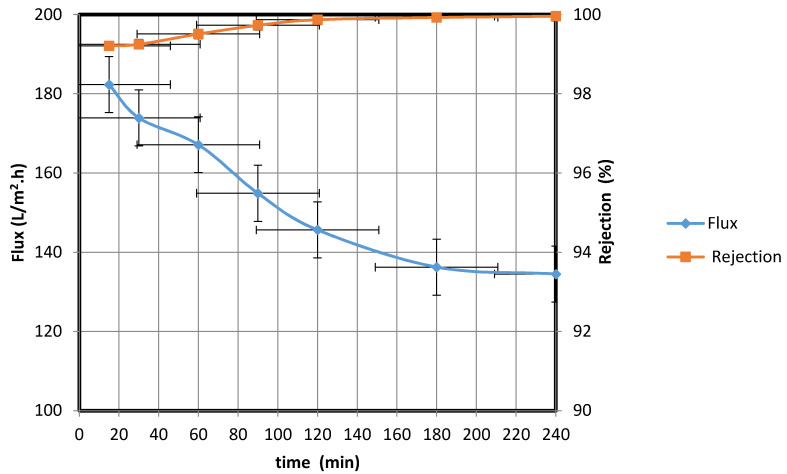
Water/bentonite clay permeate flux rate through fabricated membrane and percentage rejection of suspended materials.

The water permeate flux and rejection of pollutants at a pressure of 5 bar were then investigated using a bentonite clay suspension as the feed (870 NTU). The water permeate was collected at different intervals, and the water flux rate and percentage rejection were calculated using Equations (1) and (2), respectively (see Figure 14). Over the course of the experiment (from 15 to 240 min), the water flux rate decreased from 182.33 to 134.52 L/m^2^·h. As in the previous experiments, the decrease in the water flux rate could be attributed to the fouling of the membrane surface. The prepared membrane was found to be efficient at decreasing the turbidity of the water permeate, which reduced to 0.35 NTU by the 240th minute. These results are consistent with those obtained by Majouli et al. [14], who investigated the effectiveness of perlite membranes in terms of purifying industrial wastewater. The performance of the fabricated membrane is found to be comparable to the national wastewater discharge standards; moreover, the quality of the permeate water meets the stipulated standard (300 NTU) [28]. It is evident from the data in Figure 14 that the standard deviation is 14.07, while the mean is 162.55. The low standard deviation shows that the data are more reliable. The 90 % confidence level is 171.3, 153.81. Therefore, we are 90% confident that the true mean lies between 171.3 and 153.81; the margin of error is small.

### 3.3. Cleaning Mechanism of a Fouled Membrane and Periodic Filtration Testing

**Figure 15:**
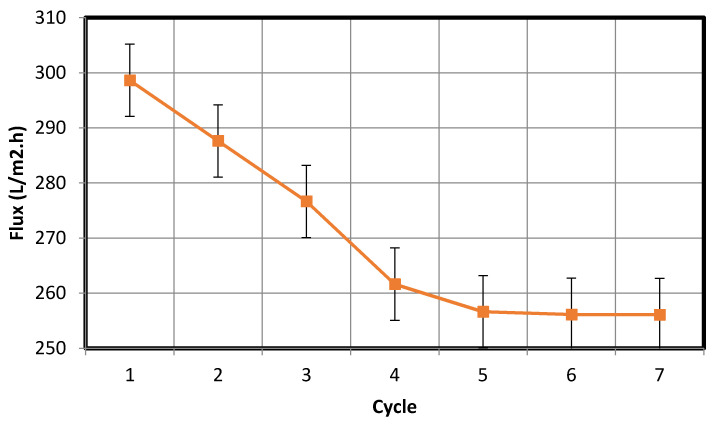
Periodic filtration for oil separation.

**Figure 16:**
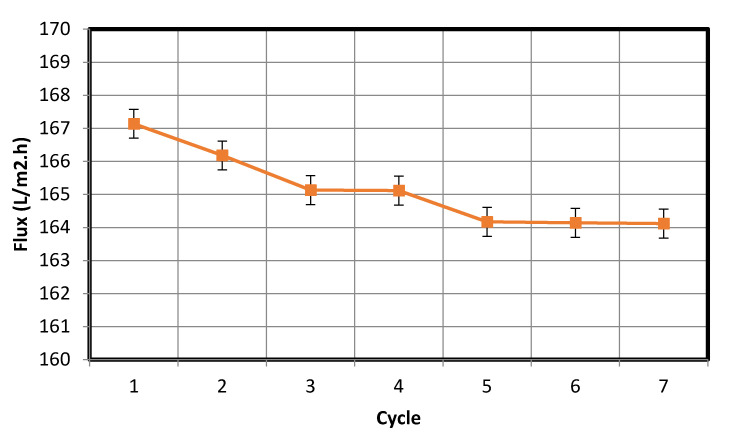
Periodic filtration for the separation of bentonite powder suspended in water.

Membrane fouling is a phenomenon that has detrimental effects on the membrane performance. During wastewater purification, fouling occurs as a result of the interaction between the membrane and particles suspended in the wastewater; the cohesion between the foulant and membrane surface depends on membrane surface properties, such as its zeta potential and hydrophilicity [22]. The intensity of fouling can be evaluated via a periodic filtration test. In this test, filtration was performed using the membrane for 60 min, after which the water permeate was collected, and the flux rate was calculated in L/m^2^·h. Backwashing was then conducted to clean the fouled membrane, and a second filtration test was performed for 60 min, following which the water permeate was collected, and the flux rate was recalculated. Overall, seven separate experimental cycles were conducted using oil/water and bentonite/water mixtures, and the results thereof are shown in Figure 15 and Figure 16, respectively. In each cycle, backwashing was executed for 10 min by mixing water with air from a compressor and passing the mixture through the membrane. As is clear from the data in Figure 15 and Figure 16, the periodic filtration cycles maintained the fabricated membrane in a condition suitable for use in industrial applications.

The flux decreases with time, the possible cause of which can be pore blockage due to deposition formation. Moreover, over the course of the filtration cycles, the degree of oil formation and material suspension on the membrane increased, which led to a reduction in water flow through the pores of the membrane and subsequently a reduced water flux rate. The fabricated membrane was used along with fine bentonite powder clay as the suspension to study the removal of the suspension from wastewater. Distilled water was used in the cleaning process and the initial turbidity was measured to be zero. Subsequently, the final turbidity of the distilled water after the cleaning process was found to be 23 NTU. This concentration of the deposited bentonite clay on the fabricated membrane is an exceedingly small amount and is equal to 2.7% of the original bentonite clay suspension concentration of 870 NTU that was used as the feed in previous experiments. Following this, we repeated the cleaning process after cleansing the membrane using fresh distilled water. At the end of the experiment, the final turbidity was found to be equal to zero. This proves the effectiveness of the proposed cleaning method to eliminate fouling. The same procedure was used to study the removal of oil emulsions from wastewater using the fabricated membrane; we found the turbidity in the used distilled water to be 8 NTU. This concentration of the deposited oil on the fabricated membrane was high and equal to 12.8% of the original ultra-pure paraffin oil concentration of 62.8 NTU. This implies that the oil deposited on the membrane (8 NTU) was removed by the suggested cleaning method.

The periodic filtration method can aid the overcoming of this tendency and maintain the flux rate at a nearly constant value owing to the repeated backwash purification of the membrane. The plots of the water flux rate as a function of cycle number in Figure 15 and Figure 16 reveal that the membrane retained a significant negative surface charge, as shown in the zeta potential plot of the fabricated membrane (Figure 9), whereby an almost constant water permeability was yielded, particularly during the suspension test (Figure 16). Moreover, this also allowed for the membrane to be efficiently cleaned. Because the membrane surface is negatively charged, it is possible to purify and reform the membrane during the backwash because the oil droplets and fine bentonite clay particles are also negatively charged; hereby, suitable water flux can be achieved under sufficient cycling. In addition, the pore size distribution of the membrane is narrow (Figure 10), leading to enhanced membrane backwashing properties. The backwashing potential arising from the repulsion between the negatively charged oil droplets and bentonite clay particles and the negatively charged membrane surface imparts upon the developed membrane qualities that make it suitable for use in water purification. As mentioned above, fouling occurs on the membrane during wastewater purification, and a mixing of distilled water with air was used to slack particulates deposited on the membrane, which allowed the discharge of the deposited materials to the feed tank. During filtration, contaminants built up on the membrane. To eliminate this, backwash by air mixed with distilled water was used. As is evident from Figure 15, the fouling is removable by the backwashes. Therefore, in Figure 15, showing the periodic filtration of oil separation, the slope of one to four cycles exhibits a steep decrease in comparison with Figure 16, showing suspension separations. This is because the oil droplets are naturally more sticky than the bentonite clay. This was also evident from the cleaning process, where more oil droplets (12.8%) were deposited on the membrane surface in comparison with the fine bentonite powder clay suspension (2.7%).

From the data in Figure 15, the standard deviation is 13.36, while the mean is 276.12. The low standard deviation implies that the data are more reliable. The 90% confidence level is (284.43, 267.81). We are 90% confident that the true mean lies between 284.43 and 267.81. The margin of error is small. In addition, from the data in Figure 16, the standard deviation is 0.873, while the mean is 165.54. The low standard deviation shows that the data are more reliable. The 90% confidence level is (166.1, 164.99). We are 90% confident that the true mean lies between 166.1 and 164.99. The margin of error is small.

## 4. Conclusions

In this study, a novel, highly effective porous membrane was developed from an inorganic material (bentonite clay and silica sand powder), incorporated with CTAB as a binder, and waste palm leaves as a pore former to create uniform pores via extrusion for the treatment of oil-in-water emulsions and fine bentonite particle/water suspensions. The developed membrane filter was tested using real oil-contaminated water obtained from the Aramco wells. The membrane was found to possess an average pore size of 66 nm, indicating that it can be used for ultrafiltration. The fabricated membranes were found to possess adequate suitable pore size distribution, and high resistance to acidic and alkaline media. The water contact angle of the fabricated membrane was determined to be 55.5°, which indicates that the membrane surface is hydrophilic. The characterization of the fabricated membrane by the three-point flexural strength technique to determine its mechanical properties revealed a sufficiently high bending strength, which was 51.53 MPa. The performance of the proposed membrane was observed to match the requirements of the national wastewater discharge standards, and the quality of the permeated water met the required standard.

The effectiveness of our fabricated membrane in terms of eliminating fouling materials during backwash can be understood in terms of the well-known mutual electrostatic repulsive property of opposite charges with identical magnitudes. The membrane could also be easily cleaned via backwashing because of the repulsion between the negatively charged oil droplets and fine bentonite particles and the negatively charged fabricated membrane surface. Overall, the results of this study indicate that the fabricated membrane is exceedingly promising for use in water purification applications.

## Figures and Tables

**Table 1:** Chemical composition (wt.%) of Saudi silica sand.

Compound	% in Silica Sand
SiO_2_	99.43
Al_2_O_3_	0.25
Fe_2_O_3_	0.031
MgO	0.024
CaO	0.16
Na_2_O	0.05
K_2_O	0.035

**Table 2:** Chemical composition (wt.%) of Saudi bentonite clay.

Compound	% in Clay
SiO_2_	55.0 ± 3.0
Al_2_O_3_	22.0 ± 2.0
TiO_2_	1.5 ± 0.25
Fe_2_O_3_	5.67 ± 0.5
MgO	2.30 ± 0.45
CaO	<2.00
Na_2_O	<2.00
K_2_O	<1.00
P_2_O_5_	<0.20
SO_3_^−^	0.002
Cl^−^	0.2
Cr_2_O_3_	0.02
Mn_2_O_3_	0.03
Loss on Ignition	9.80
